# The management concept of breast cancer with clinically node-negative/imaging node-positive disease

**DOI:** 10.1097/JS9.0000000000000664

**Published:** 2023-09-02

**Authors:** Zhao Bi, Tong-Yue Ren, Zhao-Peng Zhang, Yong-Sheng Wang

**Affiliations:** Shandong Cancer Hospital and Institute, Shandong First Medical University and Shandong Academy of Medical Sciences, Jinan, Shandong, People’s Republic of China

## Sentinel lymph node biopsy (SLNB) is suitable for patients with clinically node-negative/imaging node-positive (cN0/iN+) disease according to traditional guidelines

The increased sensitivity of axillary imaging and ultrasound (US)-guided biopsy may lead to the axilla upstaging of patients from clinically node-negative to imaging node-positive. Breast cancer patients with cN0/iN+ disease refer to patients who are considered clinically node-negative on physical examination but are found to be sonographically abnormal on imaging examination. These patients should undergo SLNB, as recommended by established guidelines. The American Society of Clinical Oncology (ASCO) guideline states that preoperative axillary US staging is not necessary for early-stage breast cancer patients in whom the sentinel lymph node (SLN) is likely to be negative (i.e. T1 and T2). In patients who are clinically node-negative on physical examination but are found to be sonographically abnormal on imaging with or without confirmatory biopsy, SLNB can be the first-line axillary staging option^1^. The National Comprehensive Cancer Network (NCCN) guideline recommends SLNB for patients with no palpable lymph node at diagnosis, or ≤2 suspicious lymph nodes on imaging, or ≤2 positive lymph nodes confirmed by needle biopsy^2^. In the era of SLNB, several randomized, controlled trials such as the ACOSOG Z0011 (American College of Surgeons Oncology Group Z0011 Trial), AMAROS (After Mapping of the Axilla: Radiotherapy or Surgery), and OTOASOR (Optimal Treatment of the Axilla – Surgery or Radiotherapy) trials showed that the rate of axillary local–regional recurrence (LRR) in patients with 1–2 positive SLNs did not change when axillary lymph node dissection (ALND) was omitted, and patients were treated with radiotherapy and adjuvant systemic therapy^3–5^. However, according to previous studies, there were ~50% of patients with cN0/iN+ disease had >2 positive lymph nodes. Therefore, for patients with cN0/iN+ disease, 50% of them could receive SLNB to omit ALND if surgery is performed first.

However, these patients need to receive regional nodal irradiation (RNI) as a supplement to axillary de-escalation surgery. The Z0011 and AMAROS trials showed that the omission of ALND, followed by radiotherapy and adjuvant systemic therapy, is safe and does not change the rate of axillary LRR among patients with limited SLN involvement^3,4^. The LRR rate is low, even in patients undergoing axillary de-escalating surgery, suggesting that tumor biology, adjuvant systemic therapy, and radiotherapy play potentially crucial roles^6^. EBCTCG (Early Breast Cancer Trialists’ Collaborative Group) meta-analyses showed that in women with node-positive breast cancer, the addition of RNI can reduce LRR, overall recurrence (any first recurrence, irrespective of whether locoregional or distant), and breast cancer mortality^7^. These findings suggest that patients with positive lymph nodes could benefit from RNI, supporting its use in patients with cN0/iN+ disease after axillary de-escalation surgery^8^.

## The widespread application of neoadjuvant therapy (NAT) could change the management of patients with cN0/iN+ disease

Patients who undergo surgery first may be upstaged to pN1 (pathological node-positive stage 1 breast cancer) status. The widespread application of NAT has contributed to optimizing the management of patients with cN0/iN+ disease. NAT is commonly used in patients with inoperable disease, as well as in those with invasive and high-risk breast cancer, such as stage II to III human epidermal growth factor receptor-2 (HER2) positive (HER2+) and triple-negative breast cancer (TNBC)^9^. Effective chemotherapy regimens, as well as targeted therapies in the neoadjuvant setting, have increased the rate of axillary pathologic complete response (apCR) after NAT, which ranges from 60 to 70% depending on the study population^9–11^. In addition, neoadjuvant endocrine therapy (NET) is increasingly being used in the treatment of hormone receptor-positive/HER2 negative (HR+/HER2−) primary tumors. The characterization and classification of breast cancer according to molecular subtypes have improved the understanding and treatment of the disease. Patients who achieve pathologic complete response (pCR) show better survival outcomes than those with residual invasive disease after NAT. A major clinical benefit of NAT is the downstaging of the tumor. In addition, it can serve as a drug sensitivity test *in vivo*, and it might prevent postoperative RNI.

### Approximately 30–40% of patients with cN0/iN+ disease may be at risk of axilla surgery up-escalated after NAT

In women with cN1 (clinical node-positive stage 1 breast cancer) breast cancer who are treated with NAT and have three or more SLNs examined, the false-negative rate (FNR) was 9.1 and 8.6% in the SENTINA (Sentinel Node vs. Axillary Dissection After Neoadjuvant Chemotherapy in Node-Positive Breast Cancer) and ACOSOG Z1071 trials, respectively^10,11^. Therefore, the 2021 St. Gallen International Consensus Guidelines’ indicated that patients with a cN1 axilla who are converted to ycN0 after NAT are potential candidates for SLNB^12^. The ASCO guideline also recommends that axillary de-escalation surgery can be achieved by placing a biopsy clip into the biopsied positive node at diagnosis and localizing it during surgery along with SLNB. In institutions in which the use of biopsy clips for nodes is not available, this can be achieved by performing SLNB with dual tracers and excising at least three SLNs to minimize the FNR and optimize the accuracy of the procedure^1^. However, patients who have residual disease after NAT, including micrometastasis, macrometastasis, and isolating tumor cells, should undergo ALND. Therefore, there might be 30–40% of patients with cN0/iN+ disease that may be at risk of axilla surgery up-escalated after NAT.

### Beneficial effects of NAT in HER2+/TNBC patients with cN0/iN+ disease

HER2+/TNBC patients with cN0/iN+ disease may benefit more from NAT than axillary de-escalation surgery. In patients with HER2+/TNBC subtype, NAT can serve as a drug sensibility test *in vivo*, which can help screen effective drugs. However, the apCR after NAT is high in these two groups of patients, which have a higher chance of preventing ALND after NAT. In patients who achieve pCR after NAT, multivariable analysis in the B04 and B41 trial populations showed that RNI was not associated with significantly improved overall survival, disease-free survival, and LRR. The RAPCHEM (Radiation After Preoperative Chemotherapy in Operable Breast Cancer) trial showed that the 5-year LRR was 2.2% (95% CI 1.4–3.4), supporting the hypothesis that it is oncologically safe to de-escalate locoregional radiotherapy based on LRR in selected patients with cT1-2N1 breast cancer treated with NAT, according to this predefined, consensus-based study guideline^13^. The 2023 St. Gallen International Consensus Guideline also states that for patients with ypN0 disease, chest wall RT and RNI can be omitted. Therefore, de-escalation of RNI after NAT is feasible for cN0/iN+ patients with ypN0 disease.

### Beneficial effects of NET in HR+/HER2− patients with cN0/iN+ disease

NET has gained increasing attention in recent years. Semiglazov *et al*.^14^. showed that the rate of breast-conserving surgery and the objective response rate is comparable between patients receiving NET and neoadjuvant chemotherapy. The 2021 St. Gallen consensus supported the use of NET in HR+/HER2− patients characterized as low-grade and/or low-genomic risk, in addition to supporting the selection of NAT (chemotherapy or endocrine therapy) strategies based on genomic tests of biopsy tissue^12^. Adjuvant chemotherapy is recommended for patients with a high residual tumor burden after NET (T>5 cm, positive residual lymph nodes), poor biological characteristics (higher grade, higher genetic risk), or those showing tumor progression during NET. The results of the POETIC (Peri-Operative Endocrine Therapy Individualising Care) trial showed that both a low initial Ki-67 value and a decrease in Ki-67 value caused by NET predict a better clinical response and prognosis^15^. This suggested that these patients were sensitive to endocrine therapy and less likely to benefit from adjuvant chemotherapy. Therefore, for patients with a good clinical response, negative lymph nodes, and a good prognosis according to PEPI (Preoperative Endocrine Prognostic Index) scoring criteria, the 2021 St. Gallen International Consensus Guidelines state that adjuvant chemotherapy can be omitted after NET^12^. The 2023 St. Gallen International Consensus Meeting also concluded that a short course of NET (2–4 weeks) before surgery and monitoring its effect on Ki-67 value can provide valuable information for waiving chemotherapy. Therefore, in HR+/HER2− patients with cN0/iN+ disease, NET is beneficial in that it can prevent the use of adjuvant chemotherapy in some patients.

Patients with cN0/iN+ disease always have a low axilla tumor burden; ~50% of them have 1–3 positive axillary lymph nodes (ALNs). Genomic tests, such as the 70-gene test (MammaPrint) and 21-gene test (Oncotype DX) could be used to inform decisions on withholding adjuvant chemotherapy in HR+/HER2− patients with 1–3 positive ALNs. However, genomic tests can only classify patients as high-risk or low-risk according to the recurrence score, and they cannot predict the sensibility of endocrine therapy. There are some patients who, although genomic tests defined as low-risk, are resistant to endocrine therapy. Therefore, NET has a better advantage than genomic tests. It can rely on the recurrence risk and individually evaluate the efficacy of endocrine therapy.

In conclusion, in patients with cN0/iN+ disease, ~50% of them could omit ALND if surgery is performed first; at the same time, RNI is necessary as a supplement to axillary de-escalation surgery. However, in HER2+/TNBC patients with cN0/iN+ disease, de-escalation of RNI is possible in those who achieve apCR after NAT. HR+/HER2− patients who show a good response after NET may not require adjuvant chemotherapy. In patients with cN0/iN+ disease, we make the following recommendations: (1) The biopsy needs to be performed guided by US to confirm the state of lymph nodes. (2) If the ALN is positive with the biopsy, patients need to receive NAT/NET first. De-escalation of RNI/chemotherapy is possible in those who achieve good response after NAT/NET. (3) If the lymph node is negative with the biopsy, the treatment strategy should be made based on molecular subtype and tumor staging. As there were 20% of patients with negative nodes have occult nodal metastases, de-escalation of RNI/chemotherapy cannot be omitted if surgery is performed first. Therefore, in clinical practice, we need to make full use of the benefits of systemic therapy and radiotherapy to reasonably reduce the scope of surgery and complications and to expand the ‘net benefit’ regarding efficacy and quality of life.

## Ethical approval

Not applicable.

## Sources of funding

Start-up fund of Shandong Cancer Hospital (Grant No. 2020PYA08), Shanghai Anti-cancer and Anti-cancer Development Foundation (CYBER-2022-002), and China Postdoctoral Science Foundation (Grant No. 2022M721987).

## Author contribution

Z.B., T.-Y.R., Z.-P.Z., and Y.-S.W.: wrote the paper. All authors read and approved the final manuscript.

## Conflicts of interest disclosure

The authors declare that they have no conflicts of interest.

## Research registration unique identifying number (UIN)

1. Name of the registry: not applicable.
2. Unique identifying number or registration ID: not applicable.
3. Hyperlink to your specific registration (must be publicly accessible and will be checked): not applicable.

## Guarantor

The guarantors are Zhao Bi and Yong-Sheng Wang.

## Data availability statement

The datasets generated during and/or analyzed during the current study are available from the corresponding author (Yong-Sheng Wang) on reasonable request. The corresponding author had full access to all the data in the study and had final responsibility for the decision to submit for publication.
